# Quantitative ultrasonographic examination of cerebral white matter by pixel brightness intensity as marker of middle-term neurodevelopment: a prospective observational study

**DOI:** 10.1038/s41598-023-44083-w

**Published:** 2023-10-05

**Authors:** Gianluigi Laccetta, Maria Di Chiara, Maria Chiara De Nardo, Monica Tagliabracci, Elisa Travaglia, Benedetta De Santis, Caterina Spiriti, Lucia Dito, Daniela Regoli, Barbara Caravale, Raffaella Cellitti, Pasquale Parisi, Gianluca Terrin

**Affiliations:** 1 Department of Maternal Infantile and Urological Sciences, Sapienza University of Rome, Rome, Italy; 2 Department of Developmental and Social Psychology, Sapienza University of Rome, Rome, Italy; 3 Department of Neuroscience, Mental Health and Sense Organs (NESMOS), Faculty of Medicine and Psychology, Sant’Andrea University Hospital, Sapienza University of Rome, Rome, Italy

**Keywords:** Biomarkers, Neurology

## Abstract

Non-cystic white matter (WM) injury has become prevalent among preterm newborns and is associated with long-term neurodevelopmental impairment. Magnetic resonance is the gold-standard for diagnosis; however, cranial ultrasound (CUS) is more easily available but limited by subjective interpretation of images. To overcome this problem, we enrolled in a prospective observational study, patients with gestational age at birth < 32 weeks with normal CUS scans or grade 1 WM injury. Patients underwent CUS examinations at 0–7 days of life (T_0_), 14–35 days of life (T_1_), 37^0/7^–41^6/7^ weeks’ postmenstrual age (T_2_), and 42^0/7^–52^0/7^ weeks’ postmenstrual age (T_3_). The echogenicity of parieto-occipital periventricular WM relative to that of homolateral choroid plexus (RE_CP_) was calculated on parasagittal scans by means of pixel brightness intensity and its relationship with Bayley-III assessment at 12 months’ corrected age was evaluated. We demonstrated that: (1) Left RE_CP_ values at T_1_ negatively correlated with cognitive composite scores; (2) Right RE_CP_ values at T_2_ and T_3_ negatively correlated with language composite scores; (3) Left RE_CP_ values at T_1_ and T_2_ negatively correlated with motor composite scores. Thus, this technique may be used as screening method to early identify patients at risk of neurodevelopmental issues and promptly initiate preventive and therapeutic interventions.

## Introduction

White matter (WM) injury (WMI) is the most frequent brain lesion in preterm newborns and may be diagnosed to some degree in up to 50% of very low birth weight (VLBW) infants^1–5^. The spectrum of WMI is composed of three major neuropathological forms: (1) Focal cystic necrosis, characterized by necrotic lesions larger than 1 mm with loss of all cellular elements; (2) Punctate WMI and focal microscopic necrosis, characterized by small necrotic foci evolving into focal areas of gliosis or microcysts smaller than 1 mm; (3) Diffuse non-necrotic lesions, characterized by changes secondary to WM dysmaturation^6^. As a consequence of the advances in neonatal care, the non-cystic form of WMI has become prevalent among preterm newborns who survive today^1,4,5,7,8^. Early diagnosis of non-cystic WM damage is crucial, given its relation with long-term neurodevelopmental impairment^2,9^. Magnetic resonance (MR) imaging (MRI) is considered the gold-standard neuroimaging method for detection of both cystic and non-cystic WMI^1^. However, this technique has some limits as it is expensive and requires both transport and sedation of the patient^1,10^. Furthermore, access to MRI is often limited, which makes serial scanning complicated^1^. On the contrary, cranial ultrasound (CUS) is a bedside tool that allows safe and reliable serial imaging; it also permits to diagnose a wide spectrum of cerebral abnormalities and to assess the evolution of injuries over time, as well as brain growth and maturation^1,11–13^. Anyway, the role of standard CUS for detection and evaluation of non-cystic WMI is modest^1^.

It has been suggested that subtle alterations in WM structure and myelination may manifest themselves as persistent periventricular hyperechogenicities and result in long-term neurodevelopmental impairment^1,14–16^. On the other side, some studies have demonstrated that periventricular densities may also reflect a benign process without consequences on neurodevelopment, such as a normal or delayed physiological maturation^17,18^. Even if CUS is a promising tool, differences in technical acquisition of images, and intra- and interobserver variability still limit the interpretation of periventricular hyperechogenicities^19,20^. As a result, attribution of a prognostic significance to these lesions by means of standard ultrasonography remains a challenge^1,9,21–23^.

In the last decades, different techniques have been developed to solve the problem of subjective interpretation of CUS scans^24–34^. These approaches include texture analysis (TA)^30–34^ and quantification of integrated backscatter (IBS)^28,29^ or pixel brightness intensity (PBI)^24–27^. However, no agreement on the preferred method of echo-quantification has been reached at present^24^. TA is difficult to apply in cases where textures occur with different scales, orientations, or translations, as it happens when characterizing biological tissues^35^. Thus, standardization of image acquisition and reconstruction parameters or postacquisition harmonization corrections are required in order to overcome this problem^35,36^. In addition, the use of a large number of radiomic metrics and the lack of uniformity of these measures and consensus regarding their selective use could lead to nonreproducible and noncomparable results^36^. Similarly, IBS needs of designated images in which the grey level is displayed proportional to the integrated backscattered power^26^. Furthermore, this technique does not solve the problem of angle-dependence of the ultrasound signal, which may decrease the diagnostic accuracy for differentiating tissue components^37^. The paucity of studies evaluating newborn brain by means of IBS reflects the lack of consensus regarding the standardization of image acquisition and analysis^28,29,38^. Software-based estimation of PBI only requires standard B-mode images^39^. This technique consists in representing the brightness of each pixel or the mean brightness of pixels within an area of interest by means of a number ranging from 0 for black to 255 for white^39^. A previous study used PBI to demonstrate that echogenicity of periventricular WM relative to bony calvarium was significantly associated with neuromotor status at term since the second week of life^24^. However, no study investigating the relationship between quantitative echogenicity of periventricular WM and middle- or long-term neurodevelopment has been conducted to date. Given the possible long-term consequences of non-cystic WMI^9^, the advantages of sequential CUS studies and the technological improvement of CUS systems^1^, the potential role of quantitative analysis of periventricular WM echogenicity in predicting middle- and long-term neurodevelopment deserves further assessment.

Starting from these premises, in the present study, we have objectively quantified periventricular WM echogenicity in a cohort of preterm infants, thus we have verified the relationship between the obtained values of echogenicity and middle-term neurodevelopment.

## Materials and methods

The study was conducted in conformity with World Medical Association Declaration of Helsinki for medical research involving human subjects. The study protocol was approved by the ethics committee of Policlinico Umberto I Hospital, Sapienza University of Rome (n° 5089). Written informed consent was obtained from parents of all newborns before recruitment.

We enrolled in a prospective observational study preterm infants with gestational age (GA) at birth < 32 weeks, consecutively admitted to the level III Neonatal Intensive Care Unit (NICU)^40^ of Policlinico Umberto I Hospital, Sapienza University of Rome, between January 1st, 2020 and December 31^st^, 2020. Enrolled patients underwent serial CUS examinations and were included in the study if they had normal CUS scans or grade I WMI in accordance with de Vries et al.^41^. Infants with WMI ≥ grade 2, subcortical WMI, grade III or IV intraventricular haemorrhage (IVH), hydrocephalus, porencephaly, cerebellar lesions, cerebral malformations, genetic syndromes, hereditary metabolic diseases, and cerebral infections were excluded.

**Figure 1:**
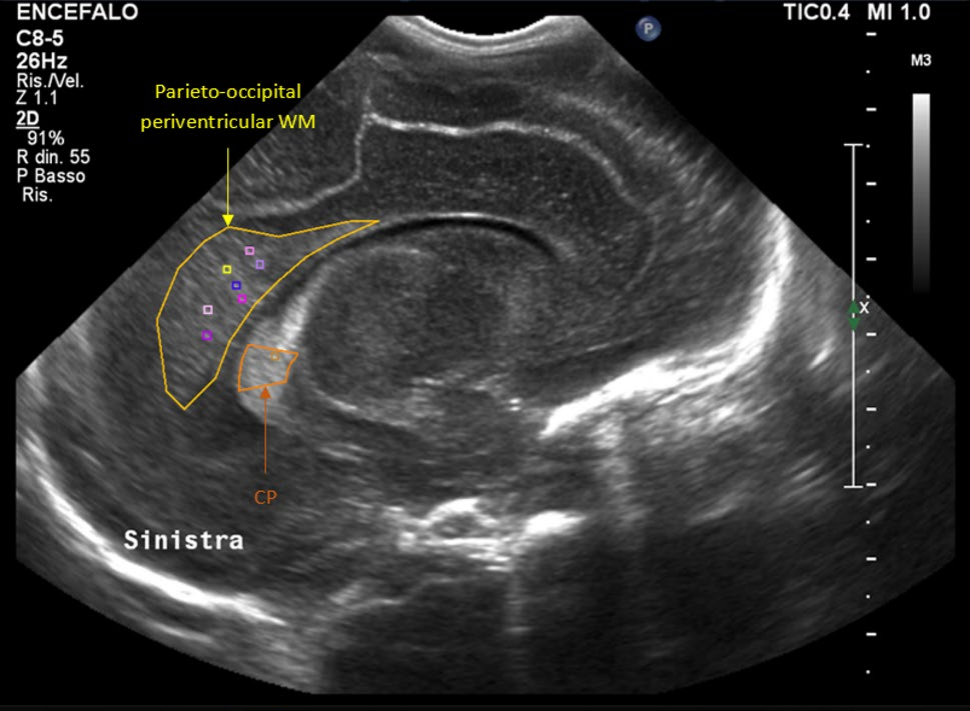
Quantitative analysis of the echogenicity of parieto-occipital periventricular WM. For details see text (“Materials and methods” section).

CUS examinations were performed at 4 different time-points: (T_0_) 0–7 days of life, (T_1_) 14–35 days of life, (T_2_) 37^0/7^–41^6/7^ weeks’ postmenstrual age (PMA), (T_3_) 42^0/7^–52^0/7^ weeks’ PMA. Protocol CUS scans were carried out by a single investigator, a neonatologist with twenty years’ experience in CUS and neonatal neuroimaging, who was unaware of the study aims. CUS examinations were performed using Affiniti 50G scanner (Philips Healthcare, Andover, MA 01810 USA) with an 8–5 MHz convex probe. No attempts were made to limit the US machine or the investigator’s ability to render the best images possible. Each CUS examination included a minimum of six standard coronal and five parasagittal views through the anterior fontanelle, and three views of the posterior fossa through the mastoid fontanelle. The sonographer selected CUS examinations belonging to infants fulfilling inclusion criteria, thus he was also responsible for the enrollment of eligible patients. A further investigator unaware of personal data and blinded regarding the clinical course of enrolled patients, selected right and left parasagittal views through the body of the lateral ventricle and performed quantitative analysis of the echogenicity of parieto-occipital periventricular WM by means of QLAB13 (Philips Andover, MA) image processing software. First of all, the anatomical borders of parieto-occipital periventricular WM and middle part of homolateral choroid plexus (CP) were manually delineated. Thus, square-shaped regions of interest (ROIs) with constant area (1.00 mm^2^) in all images were manually drawn and placed on the most hyperechoic portion of both the parieto-occipital periventricular WM and the middle part of homolateral CP. Multiple attempts to identify the most hyperechoic portion of both the parieto-occipital periventricular WM and the middle part of homolateral CP were admitted. Thus, the mean PBI (mPBI) value for each ROI was calculated. Finally, the highest mPBI value of both the parieto-occipital periventricular WM (mPBI_WMmax_) and the middle part of homolateral CP (mPBI_CPmax_) were selected. The echogenicity of parieto-occipital periventricular WM relative to that of homolateral CP (RE_CP_) was then calculated as RE_CP_ = mPBI_WMmax_/mPBI_CPmax_ (Fig. 1). Right and left RE_CP_ values were taken into account separately and were entered in a coded database for subsequent statistical analysis. CUS scans in which the parieto-occipital periventricular WM was not entirely visible were not considered for the purposes of the present study. In the meantime, a third investigator collected anamnestic details, demographics and clinical information for all enrolled newborns and entered encrypted data in the database.

A certified child neuropsychiatrist with eighteen years’ experience, who was blinded to the infants’ RE_CP_ values and unaware of the study purposes, administered the Bayley Scales of Infant and Toddler Development-Third Edition (Bayley-III) to all enrolled patients at 12 months’ corrected age (CA)^42^. Cognitive, language, and motor composite scores were used to differentiate between patients with impaired neurodevelopment (composite scores lower than 85) and patients with normal neurodevelopment (composite scores 85 or higher). Results of Bayley-III assessment have been inserted into the coded database by the child neuropsychiatrist.

Statistical Package for Social Science software (SPSS Inc., Chicago, IL, USA), version 25.0, was used to perform statistical analysis; graphs were built by means of Microsoft Excel 2021. First of all, we calculated mean with standard deviation, and percentile values of both right and left RE_CP_ at T_0_, T_1_, T_2_, and T_3_. Thus, we assumed RE_CP_ values ≥ 75th percentile as pathological and assessed the association between pathological RE_CP_ values and abnormal composite scores, and the relationship between normal RE_CP_ values and composite scores ≥ 85 by means of the χ^2^ test. For each neurodevelopmental outcome, patients were divided into cases and controls. Normality was checked by means of the Shapiro–Wilk test; mean and 95% confidence interval (CI) summarised normally distributed continuous variables. Number and percentage described categorical variables. Differences between 2 groups in the examined variables were assessed by means of the χ^2^ test for categorical variables and *t*-test or Mann–Whitney for paired and unpaired continuous variables. The correlation between RE_CP_ values and composite scores was assessed using Pearson correlation; to evaluate the influence of possible confounding factors, we performed multivariate linear regression analysis using the statistically significant variables at univariate analysis and potentially important perinatal factors as covariates. For statistical significance, we considered *p* value < 0.05. The post-hoc power based on the correlation between right RE_CP_ values at T_2_ and language composite scores at 12 months’ CA, was computed using the Post-Hoc Power Calculator available online at https://clincalc.com/stats/power.aspx with an α error probability of 0.05. The data were elaborated by a blinded statistician.

## Results

Fifty-six eligible newborns were identified. We excluded 10 patients because of post-hemorrhagic hydrocephalus (n. 2), cerebellar hemorrhage (n. 1), cystic periventricular leukomalacia alone (n. 3) or associated with cerebellar microhemorrhage (n. 1), cerebral malformations (n. 1) or infections (n. 1), and genetic syndromes (n. 1); thus, 46 patients were included for analysis. Of these, 16 (34.8%) had grade I WMI at CUS scans, whereas 30 (65.2%) had normal ultrasonographic examinations. Quantitative analysis of the echogenicity of parieto-occipital periventricular WM confirmed these results.

**Figure 2:**
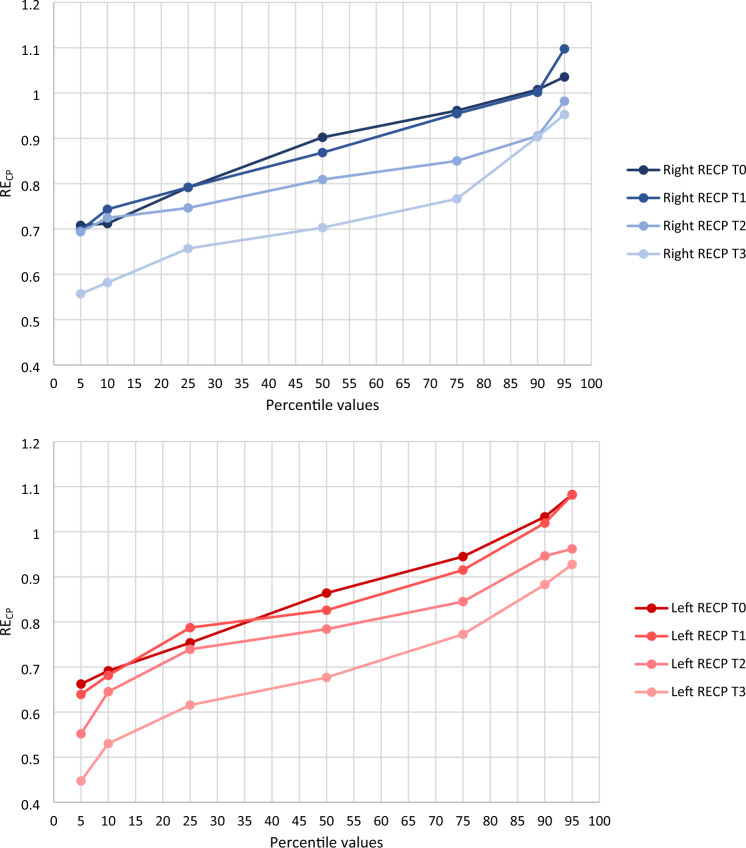
Graphical representation of right and left RE_CP_ percentile values at T_0_, T_1_, T_2_, and T_3_. Reported values are taken from Table 1. Each point represents right (upper graph) or left (lower graph) RE_CP_ value corresponding to a given percentile. For details see text (“Results” section).

**Figure 3:**
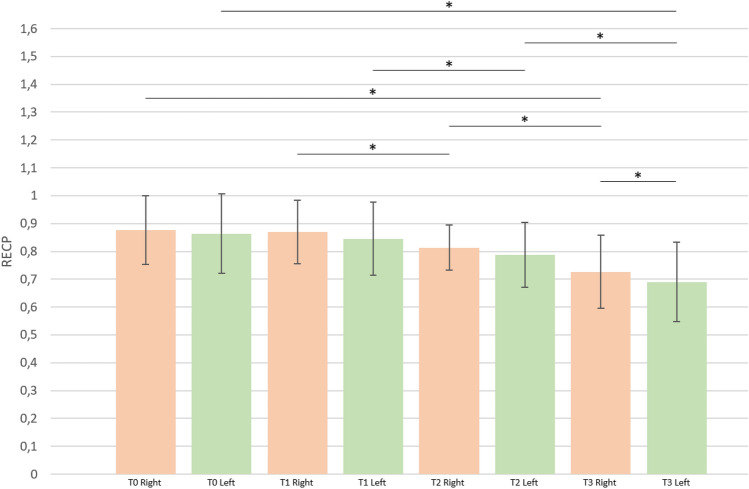
Graphical representation of mean and standard deviation of right and left RE_CP_ at T_0_, T_1_, T_2_, and T_3_; corresponding numerical values are reported in Table 1. RE_CP_, echogenicity of parieto-occipital periventricular white matter relative to that of homolateral choroid plexus; T_0_, 0–7 days of life; T_1_, 14–35 days of life; T_2_, 37^0/7^–41^6/7^ weeks’ postmenstrual age; T_3_, 42^0/7^–52^0/7^ weeks’ postmenstrual age; *statistically significant (p < 0.05).

Right and left RE_CP_ values tend to decrease with advancing postmenstrual and postnatal ages (Fig. S1). Table 1 and Fig. 2 show percentile values of both right and left RE_CP_ at T_0_, T_1_, T_2_, and T_3_ among the study participants; means with standard deviation of both right and left RE_CP_ at all 4 time points are shown in Table 1 and Fig. 3. The reported values confirm the progressive lowering of RE_CP_ over time and point out the tendency of right RE_CP_ values to be slightly higher than those calculated for the left hemisphere at the same scanning epoch; however, in this last case, the difference between right and left RE_CP_ is statistically significant only at 42^0/7^–52^0/7^ weeks’ PMA (Fig. 3).

**Table 1:** Percentile values (5th, 10th, 25th, 50th, 75th, 90th, 95th), and mean with standard deviation of right and left RE_CP_ at T_0_, T_1_, T_2_, and T_3_.

		T_0_		T_1_		T_2_		T_3_	
		Right	Left	Right	Left	Right	Left	Right	Left
RE_CP_	5th percentile	0.7081	0.6622	0.6972	0.6392	0.6940	0.5518	0.5572	0.4473
	10th percentile	0.7121	0.6916	0.7436	0.6814	0.7245	0.6453	0.5818	0.5302
	25th percentile	0.7917	0.7537	0.7920	0.7873	0.7465	0.7393	0.6571	0.6157
	**50th percentile**	**0.9023**	**0.8640**	**0.8686**	**0.8259**	**0.8092**	**0.7841**	**0.7030**	**0.6768**
	75th percentile	0.9613	0.9449	0.9545	0.9151	0.8503	0.8451	0.7666	0.7725
	90th percentile	1.0075	1.0331	1.0016	1.0193	0.9053	0.9463	0.9033	0.8829
	95th percentile	1.0355	1.0824	1.0975	1.0816	0.9821	0.9620	0.9527	0.9274
	**Mean**	**0.8771**	**0.8630**	**0.8702**	**0.8454**	**0.8138**	**0.7873**	**0.7264**	**0.6901**
	SD	0.1227	0.1428	0.1139	0.1307	0.0805	0.1162	0.1310	0.1428

The 50th percentile is the value such that half of the sample is below and the other half is above; it corresponds to the median value of the sample. Percentile values were calculated by means of the SPSS 25.0 Empirical distribution function; in this case, the percentile value is *X*_*i*_, where *i* is equal to *wp* rounded up to the next integer (*w* is the sum of the weights for all nonmissing cases, *p* is the specified percentile divided by 100).

RE_CP_, echogenicity of parieto-occipital periventricular white matter relative to homolateral choroid plexus; SD, standard deviation; T_0_, 0–7 days of life; T_1_, 14–35 days of life; T_2_, 37^0/7^–41^6/7^ weeks’ postmenstrual age; T_3_, 42^0/7^–52^0/7^ weeks’ postmenstrual age.

The main prenatal, perinatal, and clinical characteristics of the study population, in relation to the neurodevelopmental outcomes at 12 months’ CA, are shown in Table 2. Similarly, morbidities during hospital stay of the study population are represented in Table 3.

**Table 2:** Characteristics of the study population in relation to neurodevelopmental outcomes at 12 months’ CA.

	Cognitive scale at 12 months’ CA		Language scale at 12 months’ CA		Motor scale at 12 months’ CA	
	Cases^a^	Controls^b^	Cases^a^	Controls^b^	Cases^a^	Controls^b^
Patients, *N (%)*	3 (6.5)	43 (93.5)	7 (15.6)	38 (84.4)	9 (20.0)	36 (80.0)
Maternal age, *years*	33.33 (19.21–47.46)	33.60 (32.41–34.80)	33.71 (29.76–37.66)	33.50 (32.19–34.81)	32.56 (30.00–35.11)	33.78 (32.38–35.17)
Pregnancy-induced pressure disorders, *N (%)*	1 (33.3)	8 (18.6)	2 (28.6)	7 (18.4)	3 (33.3)	6 (16.7)
Maternal thyroid disorders, *N (%)*	1 (33.3)	5 (11.6)	2 (28.6)	4 (10.5)	1 (11.1)	5 (13.9)
Alterations of Doppler flow velocimetry^c^, *N (%)*	0 (0.0)	8 (18.6)	0 (0.0)	8 (21.1)	2 (22.2)	6 (16.7)
IUGR, *N (%)*	0 (0.0)	5 (11.6)	0 (0.0)	5 (13.2)	0 (0.0)	5 (13.9)
Twin infants, *N (%)*	0 (0.0)	9 (20.9)	1 (14.3)	8 (21.1)	1 (11.1)	8 (22.2)
Antenatal corticosteroids^d^, *N (%)*	2 (66.7)	33 (76.7)	5 (71.4)	30 (78.9)	8 (88.9)	27 (75.0)
Cesarean section, *N (%)*	2 (66.7)	40 (93.0)	6 (85.7)	35 (92.1)	7 (77.8)	34 (94.4)
Gestational age, *weeks*	25.33 (20.16–30.50)*	29.77 (29.14–30.40)*	27.14 (24.79–29.50)*	29.84 (29.18–30.51)*	27.78 (25.58–29.98)*	29.83 (29.16–30.50)*
ELBW, *N (%)*	1 (33.3)	8 (18.6)	2 (28.6)	7 (18.4)	3 (33.3)	6 (16.7)
SGA, *N (%)*	0 (0.0)	6 (14.0)	1 (14.3)	5 (13.2)	1 (11.1)	5 (13.9)
Male sex, *N (%)*	2 (66.7)	21 (48.8)	6 (85.7)	17 (44.7)	5 (55.6)	18 (50.0)
5-min Apgar score	7.33 (6.54–8.13)	8.09 (7.81–8.38)	7.86 (6.73–8.98)	8.08 (7.77–8.39)	7.78 (7.03–8.52)	8.11 (7.78–8.44)
Umbilical cord arterial pH	7.41 (7.10–7.73)*	7.27 (7.25–7.29)*	7.30 (7.21–7.40)	7.27 (7.25–7.30)	7.31 (7.25–7.38)	7.27 (7.24–7.29)
Body temperature at 1 h of life, *°C*	35.75 (32.57–38.93)	36.24 (36.08–36.39)	36.08 (35.72–36.44)	36.23 (36.05–36.41)	36.02 (35.68–36.37)	36.25 (36.07–36.43)
Recovery of birth weight within 14 days of life, *N (%)*	2 (66.7)	27 (65.9)	5 (71.4)	24 (66.7)	5 (55.6)	24 (70.6)
Duration of parenteral nutrition, *days*	30.67 (16.62–77.95)*	12.63 (8.87–16.38)*	26.29 (11.86–40.71)*	11.87 (8.04–15.69)*	20.33 (8.68–31.99)	12.56 (8.34–16.77)
Length of hospital stay, *days*	88.00 (56.97–119.03)*	55.21 (47.75–62.67)*	77.57 (54.25–100.89)*	54.03 (46.26–61.80)*	75.67 (49.29–102.04)*	53.19 (46.26–60.13)*

As expected, GA at birth was lower in cases with respect to controls. On the contrary, arterial cord blood pH was lower in patients with normal cognitive composite score at 12 months’ CA. The duration of parenteral nutrition (PN) was longer in infants with cognitive or language impairment; the length of hospital stay was longer in patients with pathological cognitive, language, or motor composite scores at Bayley-III assessment.

Mean and 95% confidence interval summarise normally distributed continuous variables; number and percentage describe categorical variables.

*p < 0.05.

^a^Composite score < 85.

^b^Composite score ≥ 85.

^c^Umbilical artery.

^d^Intramuscular steroid cycle in two doses of 12 mg over a 24 h period; *CA* corrected age, *ELBW* extremely low birth weight, *IUGR* intrauterine growth restriction, *SGA* small for gestational age.

**Table 3:** Morbidity during hospital stay of the study population in relation to neurodevelopmental outcomes at 12 months’ CA.

	Cognitive scale at 12 months’ CA		Language scale at 12 months’ CA		Motor scale at 12 months’ CA	
	Cases^a^	Controls^b^	Cases^a^	Controls^b^	Cases^a^	Controls^b^
Patients, *N (%)*	3 (6.5)	43 (93.5)	7 (15.6)	38 (84.4)	9 (20.0)	36 (80.0)
Necrotising enterocolitis, *N (%)*	0 (0.0)	4 (9.3)	0 (0.0)	4 (10.5)	0 (0.0)	4 (11.1)
Bronchopulmonary dysplasia, *N (%)*	0 (0.0)	1 (2.3)	0 (0.0)	1 (2.6)	1 (11.1)	0 (0.0)
Culture-positive sepsis, *N (%)*	1 (33.3)	2 (4.7)	2 (28.6)	1 (2.6)	1 (11.1)	2 (5.6)
Retinopathy of prematurity, *N (%)*	2 (66.7)*	3 (7.0)*	2 (28.6)	3 (7.9)	2 (22.2)	3 (8.3)
Patent ductus arteriosus^c^, *N (%)*	2 (66.7)	6 (14.0)	2 (28.6)	6 (15.8)	4 (44.4)*	4 (11.1)*
Anemia of prematurity, *N (%)*	2 (66.7)	7 (16.3)	3 (42.9)	6 (15.8)	4 (44.4)	5 (13.9)
Metabolic complications^d^, *N (%)*	2 (66.7)	10 (23.3)	3 (42.9)	9 (23.7)	3 (33.3)	9 (25.0)

The incidences of retinopathy of prematurity (ROP) and hemodynamically significant (hs) patent ductus arteriosus (hsPDA) were higher in patients with abnormal cognitive and motor composite scores at 12 months’ CA, respectively.

*p < 0.05.

^a^Composite score < 85.

^b^Composite score ≥ 85.

^c^Hemodynamically significant.

^d^Hypo/hypernatremia, hypo/hyperkalemia, hypo/hypercalcemia, hypo/hyperphosphatemia, hypo/hypermagnesemia, hypo/hyperchloremia, hypo/hyperglycemia, hypertryglyceridemia, metabolic acidosis; *CA* corrected age.

As regards the role of RE_CP_ values in predicting neurodevelopmental outcomes at 12 months’ CA, we observed that left RE_CP_ values greater than or equal to the 75th percentile at 37^0/7^–41^6/7^ weeks’ PMA permitted to identify all patients with pathological cognitive composite scores (Table S1, Supplementary material). Similarly, right RE_CP_ values corresponding to the 75th percentile after 14 days of life and left and right RE_CP_ values corresponding to the 75th percentile at term-equivalent age (TEA) allowed to successfully distinguish between patients with normal and pathological language composite scores (Table S2, Supplementary material). Finally, left RE_CP_ values corresponding to the 75th percentile after 14 days of life and right RE_CP_ values corresponding to the 75th percentile at 14–35 days of life and 42^0/7^–52^0/7^ weeks’ PMA permitted to properly differentiate patients based on their middle-term motor outcome (Table S3, Supplementary material).

Correlations between RE_CP_ values from both right and left parasagittal scans at the scheduled time points (T_0_, T_1_, T_2_ and T_3_) and neurodevelopmental composite scores at 12 months’ CA are shown in Table S4 (Supplementary material) and Figs. S2–S4 (Supplementary material). No correlation between RE_CP_ values in the first week of life and neurodevelopmental composite scores was demonstrated (Table S4, Supplementary material).

The results of multivariate linear regression analysis between RE_CP_ values at T_1_, T_2_ and T_3_, and neurodevelopmental composite scores at 12 months’ CA are shown in Table 4. According to our multivariate linear regression analysis, RE_CP_ values from the left parasagittal scan at 14–35 days of life negatively correlated with cognitive composite scores at 12 months’ CA (Table 4). As regards language development, RE_CP_ values from the right parasagittal scan after 37 weeks 0 days’ PMA negatively correlated with composite scores at 12 months’ CA (Table 4). Multivariate linear regression analysis also demonstrated that RE_CP_ values from the left parasagittal scan at 14–35 days of life and 37^0/7^–41^6/7^ weeks’ PMA negatively correlated with motor composite scores at 12 months’ CA; a similar but borderline trend (p = 0.055) was observed for right RE_CP_ values at 14–35 days of life (Table 4).

**Table 4:** Multivariate linear regression analysis between RE_CP_ values at T_1_, T_2_ and T_3_, and neurodevelopmental composite scores at 12 months’ CA.

				Covariates					
				Gestational Age	Arterial pH^a^	Duration of PN	Lenght of HS	ROP	hsPDA
Cognitive composite score at 12 months’ CA	T_1_	Right RE_CP_	β = − 0.300 (p = 0.067)	β = 0.170 (p = 0.325)	β = − 0.123 (p = 0.421)	β = − 0.068 (p = 0.686)			
		Left RE_CP_	*β* = *− 0.394 (p* = 0.022)*	β = 0.139 (p = 0.411)	β = − 0.038 (p = 0.804)	β = − 0.048 (p = 0.769)			
	T_2_	Right RE_CP_	β = 0.307 (p = 0.066)	β = − 0.394 (p = 0.094)	β = 0.253 (p = 0.118)	β = 0.103 (p = 0.577)	β = − 0.244 (p = 0.320)	β = 0.255 (p = 0.148)	
		Left RE_CP_	β = 0.239 (p = 0.154)	β = − 0.397 (p = 0.101)	β = 0.218 (p = 0.192)	β = 0.141 (p = 0.452)	β = − 0.231 (p = 0.359)	β = 0.236 (p = 0.192)	
	T_3_	Right RE_CP_	β = − 0.238 (p = 0.212)	β = 0.068 (p = 0.789)	β = − 0.099 (p = 0.569)	β = 0.011 (p = 0.957)	β = − 0.163 (p = 0.564)	β = − 0.112 (p = 0.559)	
		Left RE_CP_	β = − 0.350 (p = 0.078)	β = 0.023 (p = 0.926)	β = − 0.117 (p = 0.488)	β = 0.042 (p = 0.829)	β = − 0.133 (p = 0.626)	β = − 0.096 (p = 0.606)	
Language composite score at 12 months’ CA	T_1_	Right RE_CP_	β = − 0.207 (p = 0.191)	β = 0.239 (p = 0.160)	β = − 0.078 (p = 0.602)	β = − 0.209 (p = 0.209)			
		Left RE_CP_	β = − 0.080 (p = 0.637)	β = 0.265 (p = 0.131)	β = − 0.065 (p = 0.682)	β = − 0.232 (p = 0.174)			
	T_2_	Right RE_CP_	*β* = *− 0.609 (p* = 0.000)*	β = − 0.349 (p = 0.103)	β = 0.068 (p = 0.623)	β = 0.094 (p = 0.581)	β = − 0.154 (p = 0.494)		
		Left RE_CP_	β = 0.347 (p = 0.063)	β = − 0.353 (p = 0.171)	β = 0.008 (p = 0.961)	β = 0.202 (p = 0.327)	β = − 0.126 (p = 0.644)		
	T_3_	Right RE_CP_	*β* = *− 0.386 (p* = 0.024)*	β = 0.156 (p = 0.482)	β = − 0.082 (p = 0.575)	β = − 0.084 (p = 0.632)	β = − 0.132 (p = 0.595)		
		Left RE_CP_	β = − 0.092 (p = 0.630)	β = 0.084 (p = 0.724)	β = − 0.088 (p = 0.577)	β = − 0.139 (p = 0.468)	β = − 0.277 (p = 0.304)		
Motor composite score at 12 months’ CA	T_1_	Right RE_CP_	β = − 0.308 (p = 0.055)	β = 0.142 (p = 0.408)	β = − 0.018 (p = 0.908)				β = − 0.218 (p = 0.190)
		Left RE_CP_	*β* = *− 0.448 (p* = 0.011)*	β = 0.143 (p = 0.378)	β = 0.065 (p = 0.668)				β = − 0.075 (p = 0.652)
	T_2_	Right RE_CP_	β = 0.268 (p = 0.149)	β = − 0.045 (p = 0.873)	β = 0.155 (p = 0.387)		β = 0.168 (p = 0.532)		β = 0.243 (p = 0.214)
		Left RE_CP_	*β* = *− 0.437 (p* = 0.023)*	β = − 0.104 (p = 0.691)	β = 0.102 (p = 0.545)		β = 0.132 (p = 0.599)		β = 0.082 (p = 0.666)
	T_3_	Right RE_CP_	β = − 0.152 (p = 0.405)	β = − 0.003 (p = 0.991)	β = − 0.027 (p = 0.870)		β = − 0.265 (p = 0.322)		β = − 0.217 (p = 0.229)
		Left RE_CP_	β = − 0.159 (p = 0.442)	β = − 0.017 (p = 0.946)	β = − 0.037 (p = 0.822)		β = − 0.264 (p = 0.331)		β = − 0.183 (p = 0.342)

CUS examination at TEA resulted the most predictive of middle-term neurodevelopment (β = − 0.609 for the correlation between right RE_CP_ values and language composite scores; β = − 0.437 for the correlation between left RE_CP_ values and motor composite scores).

^a^Sample taken from umbilical cord; CA, corrected age; CUS, cranial ultrasound; HS, hospital stay; hsPDA, hemodynamically significant patent ductus arteriosus; PN, parenteral nutrition; RE_CP_, echogenicity of parieto-occipital periventricular white matter relative to homolateral choroid plexus; ROP, retinopathy of prematurity; *TEA* term-equivalent age; T_1_; 14–35 days of life; T_2_, 37^0/7^–41^6/7^ weeks’ postmenstrual age; T_3_, 42^0/7^–52^0/7^ weeks’ postmenstrual age; *statistically significant (p < 0.05).

Post-hoc analysis demonstrated a power of 97.9% to detect the observed differences in right RE_CP_ at TEA between patients with pathological and normal language composite score at 12 months’ CA.

## Discussion

Quantitative analysis of CUS scans by means of PBI and measurement of the echogenicity of parieto-occipital periventricular WM relative to that of homolateral CP (RE_CP_) enable us to overcome the problem of differences in technical acquisition of images, and operator-dependent variability in the interpretation of periventricular hyperechogenicities. Thus, the aforementioned technique could increase the opportunities of CUS to properly classify WMI and detect patients at risk of neurodevelopmental sequelae who deserve early neuroprotective and rehabilitative interventions while waiting for a more precise characterization of brain damage through MRI.

We employed PBI for quantitative assessment of the echogenicity of periventricular WM because of its ease of use, which may facilitate the widespread diffusion of this technique in the clinical context. Previous studies aimed at evaluating the quantitative echogenicity of neonatal brain parenchyma by means of PBI^24–27,43^, but only one evaluated the prognostic value of this approach^24^. In this study, the relative echogenicity of periventricular WM was compared with neurodevelopmental outcome evaluated by means of the Lacey Assessment of the Preterm Infant (LAPI) at TEA^24^. Coronal scans were acquired without standardization of parameters in the first week of life, at 2–5 weeks of life and beyond the sixth week of life in patients with GA at birth < 34 weeks; thus, mPBI was calculated within circular ROIs of constant size in the fronto-parietal and parieto-occipital regions^24^. The relative echogenicity of fronto-parietal periventricular WM in relation to CP was negatively correlated with PMA and postnatal age^24^; in our study, the same relationship was demonstrated for the relative echogenicity of parieto-occipital periventricular WM in relation to CP. Differently from the study by Beller et al.^24^, we demonstrated the effectiveness of this technique in early prediction of middle-term neurodevelopment. In particular, we pointed out the relationship between the echogenicity of parieto-occipital periventricular WM, relative to that of homolateral CP, and Bayley-III neurodevelopmental composite scores at 12 months’ CA. Similarly to Beller et al., we did not demonstrate any significant association between the relative echogenicity of periventricular WM in the first week of life and later neurodevelopment^24^. We think a growing body of medical research could explain this finding. Periventricular hyperechogenicity is a common finding in preterm infants and its persistence for more than 7 days constitutes an important risk factor for a potential evolution of this lesion^6,44^. In addition, the first ultrasonographic equivalent of hypoxic-ischemic brain damage occurring in the perinatal period is constituted by the appearance of periventricular hyperechogenicity which, however, does not take place before 3 days of life^44^. Thus, CUS examination performed in the first week of life may have a reduced sensitivity in detecting cerebral insults, especially if carried out in the first 3 days of life; at the same time, specificity could be reduced since periventricular hyperechogenicity, within the first week of life, can take on a transitory character. Preterm birth probably causes reduced or delayed expression of antioxidant enzymes in oligodendrocyte precursor cells which may become more susceptible to oxidative damage and undergo necrosis or apoptosis^45^. The presence of reactive oxygen species also induces the proliferation of oligodendrocyte precursor cells that are unable to produce myelin^45^. Thus, the damage of WM gives rise to structural reorganization and subsequent long-term neurodevelopmental impairment^45^. These processes, however, require time to be established^45^ and early CUS examination may not detect changes in the echogenicity of periventricular WM and be predictive of later neurodevelopment; this was also the reason for which we decided to analyse CUS scans until 42^0/7^–52^0/7^ weeks’ PMA.

Beller et al. demonstrated that a significant negative correlation between motor, neurological and developmental LAPI scores and echogenicity of fronto-parietal periventricular WM in comparison with BN was already present at 2–5 weeks of life; the same authors also pointed out a significant negative correlation between motor and developmental LAPI scores and relative echogenicity of parieto-occipital periventricular WM in comparison with BN beyond the sixth week of life^24^. In our study, we focused on the echogenicity of parieto-occipital periventricular WM, since it matures before the fronto-parietal periventricular WM^46^, thus permitting to raise suspicion of neurodevelopmental impairment earlier than the examination of anterior periventricular WM, and proved that its negative correlation with middle-term neurodevelopment was present since the 14th day of life. As denominator, we chose the intermediate portion of CP, considered that this one is the most hyperechoic area of this brain region^25^ and CP is used by most clinicians as reference for qualitative assessment of the echogenicity of periventricular WM^24,41^. After some instability in the first days of life, related to ventilation, haemorrhages or hypoxic-ischemic insults, CP has a relatively constant blood flow and echogenicity^24,25,47^; however, the echogenicity of CP may not be superimposable between the two sides. We hypothesized that discrepancies in cerebral blood flow between the two hemispheres, favoured by the different origin of common carotid arteries (brachiocephalic artery on the right, aortic arch on the left)^48^, may explain this assumption and even side differences in brain maturation (as shown by the progressive lowering of RE_CP_ values over time and statistically significant difference between right and left RE_CP_ at T_3_) and distribution of cerebral functions. Indeed, we showed an association between cognitive and motor skills and left hemisphere of the brain, whereas language could be right-sided in the early stages of life; these findings are even in accordance with previous studies^46,49^. Some authors demonstrated that asymmetries of brain functions are evident since 12–27 weeks’ GA by observing foetuses’ and neonates’ movements and gene expression in embryos, and constitute a dynamic fact as the right and left predominance of these functions evolves physiologically over the course of lifetime^50^. We hypothesize that topographic differences in brain maturation early on may render certain part of the brain, that carries certain functions, more susceptible to injury and adverse outcomes. So, given that left hemisphere is maturing faster, it could be more susceptible to injury. However, further studies are necessary to demonstrate this hypothesis.

Despite being interesting, our results should be interpreted considering some limitations. First of all, our study is conditioned by the small number of included patients. A greater amount of infants would have allowed to assess the accuracy of RE_CP_, measured by means of PBI, in predicting middle-term neurodevelopment, and even the impact of different gestational ages on results of quantitative analysis of the echogenicity of parieto-occipital periventricular WM. However, post-hoc analysis demonstrated that sample size was large enough not to affect the main outcome of our research. Second, we used Bayley-III testing at 12 months’ CA, which could be relatively early to assess neurodevelopment in preterm-born children, especially cognition and language^51^. However, Krogh et al. examined the predictive validity of all scales and subtests from Bayley-III in a cohort of 55 children who were assessed at six time points between 4 and 36 months^52^. The authors found significant correlations for all ages and scales/subtests; in particular, cognitive scale was predictive at 7–13 months, language scale was predictive from 10 months, and motor scale was predictive at all ages but only with respect to adjacent and not distant assessment points^52^. Furthermore, Klein-Radukic et al. investigated the predictive value of the cognitive scale of Bayley-III at the ages of 6,9,18, and 26 months; thus, the authors concluded that Bayley-III at all ages predicted the children’s intelligence quotient obtained using the Wechsler Preschool and Primary Scale of Intelligence-Third Edition at 50 months, and all Bayley-III scores predicted subsequent Bayley-III scores^53^. Furthermore, we were interested in early identification of patients at risk of neurodevelopmental issues: for this reason, we only investigated the relationship between WM echogenicity and middle-term neurodevelopment. However, the relationship between RE_CP_ at different time points and long-term neurodevelopment (18–36 months of age) surely deserves further assessment in future studies. In addition, both operators carrying out CUS scans and Bayley-III assessment were aware of the patient’s clinical condition and medical history; however, this limit was difficult to eliminate because performing both examinations would have required interaction with the baby and knowledge of anamnesis. Anyway, both the sonographer and the child neuropsychiatrist were unaware about the study purposes. Furthermore, a third-party observer was involved in data collection and a blinded statistician performed data analysis in order to reduce the risk of assessment bias. Finally, a technical issue is represented by the modification of the acoustic window with advancing age: in rare cases, the anterior fontanelle becomes so small that a proper examination of periventricular WM is not possible. However, in our study, we excluded CUS scans in which periventricular WM was not fully appreciable.

## Conclusion

The results of this study could lead to important implications in clinical practice. Given that CUS is simple, risk-free and repeatable, the quantitative analysis of periventricular WM echogenicity may be used as a screening method to early identify patients at risk of neurodevelopmental issues and promptly initiate preventive and therapeutic interventions.

### Supplementary Information

Supplementary Figure S1.

Supplementary Figure S2.

Supplementary Figure S3.

Supplementary Figure S4.

Supplementary Table S1.

Supplementary Table S2.

Supplementary Table S3.

Supplementary Table S4.

## Data Availability

The dataset used and analysed during the current study is available from the corresponding author on reasonable request.
