# Supplementary material for: Quantitative ultrasonographic examination of cerebral white matter by pixel brightness intensity as marker of middle-term neurodevelopment: a prospective observational study
Source: Sci Rep. 2023 Oct 5;13:16816. doi: 10.1038/s41598-023-44083-w (PMC10556025; doi:10.1038/s41598-023-44083-w)
Supplement: Supplementary file 1 — Supplementary Figure S1. [file 41598_2023_44083_MOESM1_ESM.docx]

Figure S1. Maturation process of right and left periventricular WM according to postmenstrual age (upper graph) and postnatal age (lower graph), with progressive lowering of RE_CP_ values
