# Supplementary material for: Quantitative ultrasonographic examination of cerebral white matter by pixel brightness intensity as marker of middle-term neurodevelopment: a prospective observational study
Source: Sci Rep. 2023 Oct 5;13:16816. doi: 10.1038/s41598-023-44083-w (PMC10556025; doi:10.1038/s41598-023-44083-w)
Supplement: Supplementary file 2 — Supplementary Figure S2. [file 41598_2023_44083_MOESM2_ESM.docx]

**
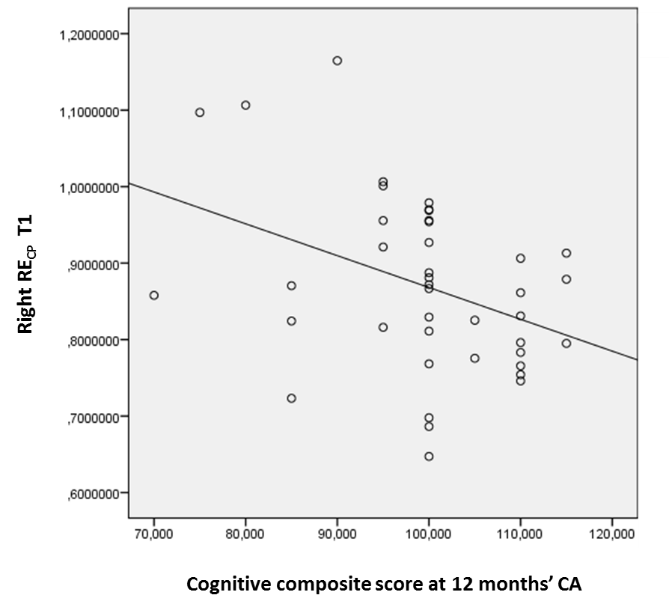

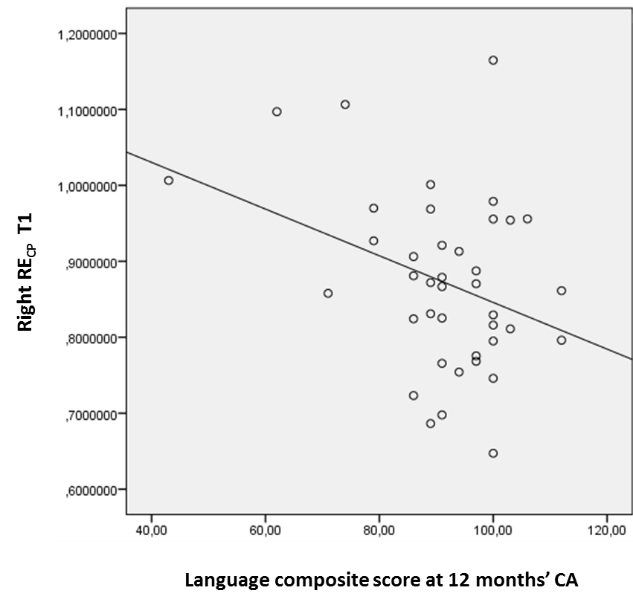
**


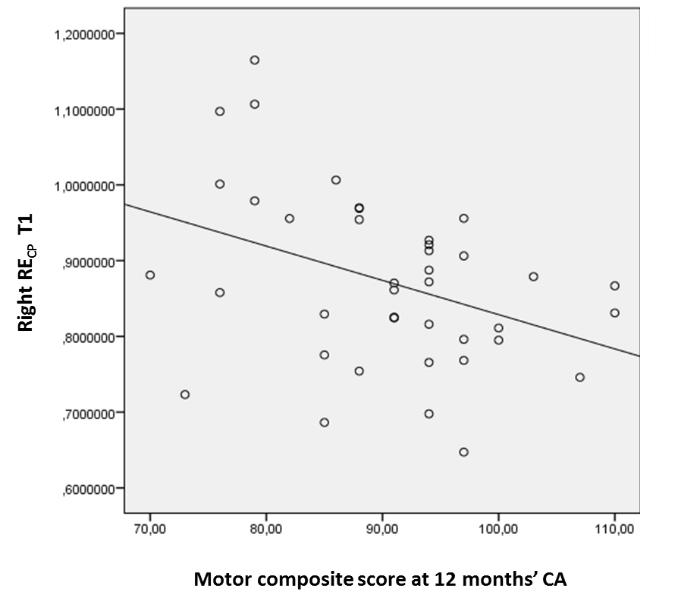

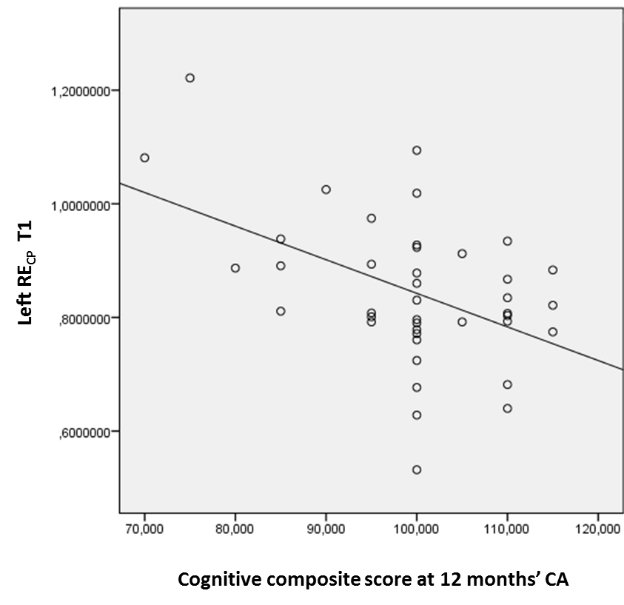


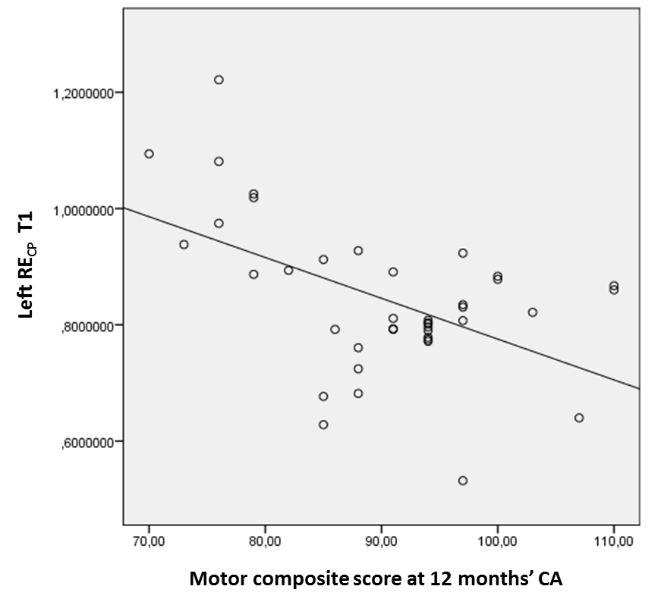


Figure S2. Significant correlations between RE_CP_ values from both right and left parasagittal scans at T_1_ and neurodevelopmental composite scores at 12 months’ CA. Cognitive composite score: Right RE_CP_ (*r*= -0.380, *p*=0.014) and Left RE_CP_ (*r*= -0.471, *p*=0.002). Language composite score: Right RE_CP_ (*r*= -0.345, *p*=0.029). Motor composite score: Right RE_CP_ (*r*= -0.382, *p*=0.015) and Left RE_CP_ (*r*= -0.515, *p*=0.001).
