# Supplementary material for: Quantitative ultrasonographic examination of cerebral white matter by pixel brightness intensity as marker of middle-term neurodevelopment: a prospective observational study
Source: Sci Rep. 2023 Oct 5;13:16816. doi: 10.1038/s41598-023-44083-w (PMC10556025; doi:10.1038/s41598-023-44083-w)
Supplement: Supplementary file 3 — Supplementary Figure S3. [file 41598_2023_44083_MOESM3_ESM.docx]

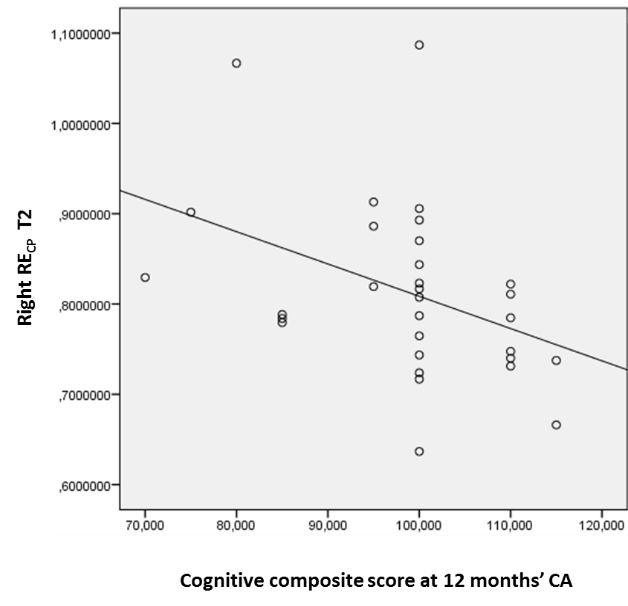
 **
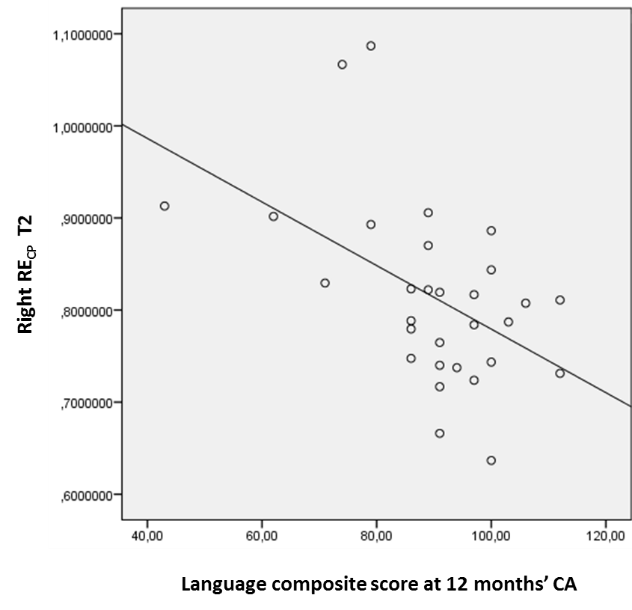
**

**
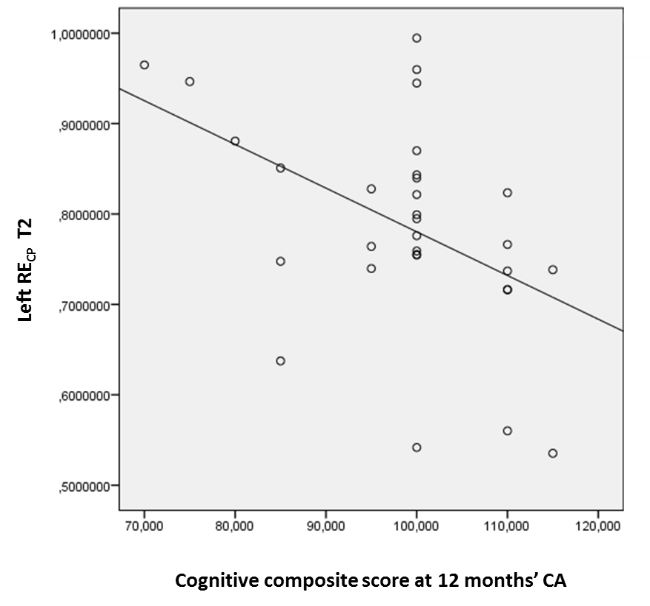
**
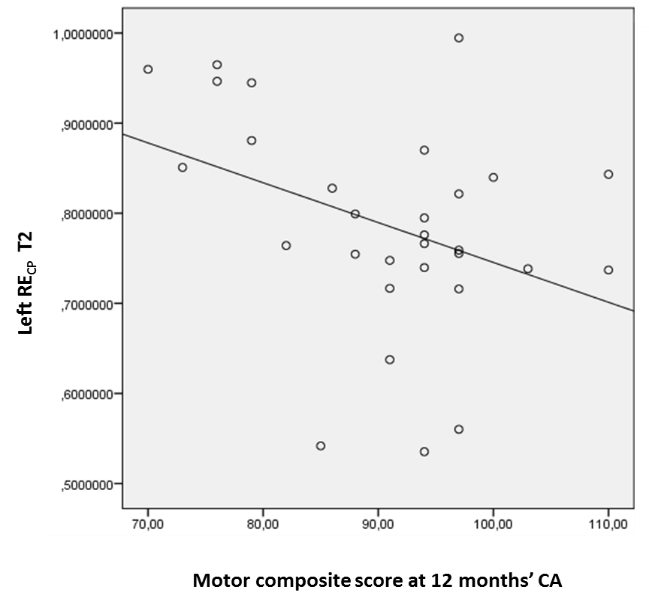


Figure S3. Significant correlations between RE_CP_ values from both right and left parasagittal scans at T_2_ and neurodevelopmental composite scores at 12 months’ CA. Cognitive composite score: Right RE_CP_ (*r*= -0.405, *p*=0.024) and Left RE_CP_ (*r*= -0.460, *p*=0.009). Language composite score: Right RE_CP_ (*r*= -0.496, *p*=0.005). Motor composite score: Left RE_CP_ (*r*= -0.373, *p*=0.042).
