# Supplementary material for: Quantitative ultrasonographic examination of cerebral white matter by pixel brightness intensity as marker of middle-term neurodevelopment: a prospective observational study
Source: Sci Rep. 2023 Oct 5;13:16816. doi: 10.1038/s41598-023-44083-w (PMC10556025; doi:10.1038/s41598-023-44083-w)
Supplement: Supplementary file 4 — Supplementary Figure S4. [file 41598_2023_44083_MOESM4_ESM.docx]

**
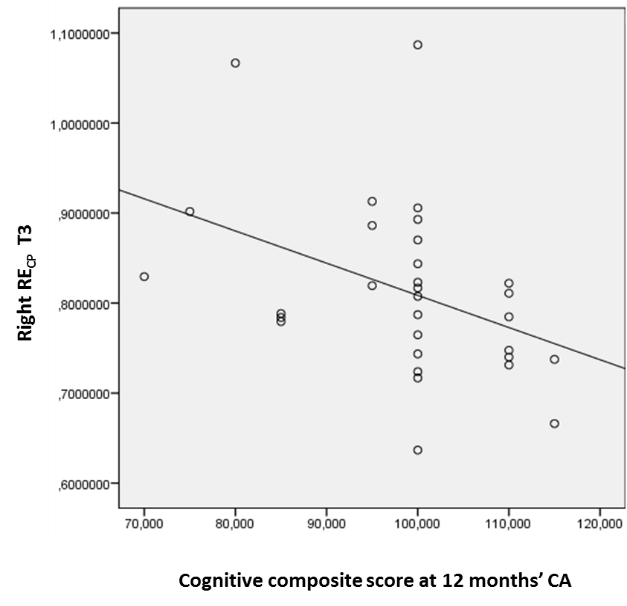

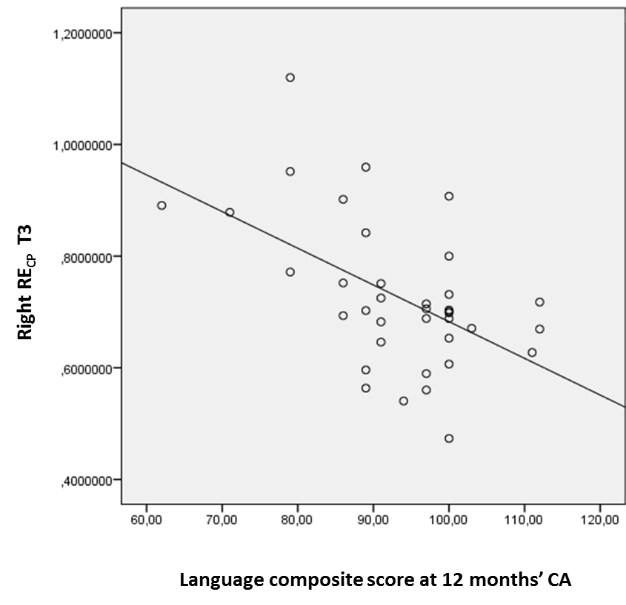
**


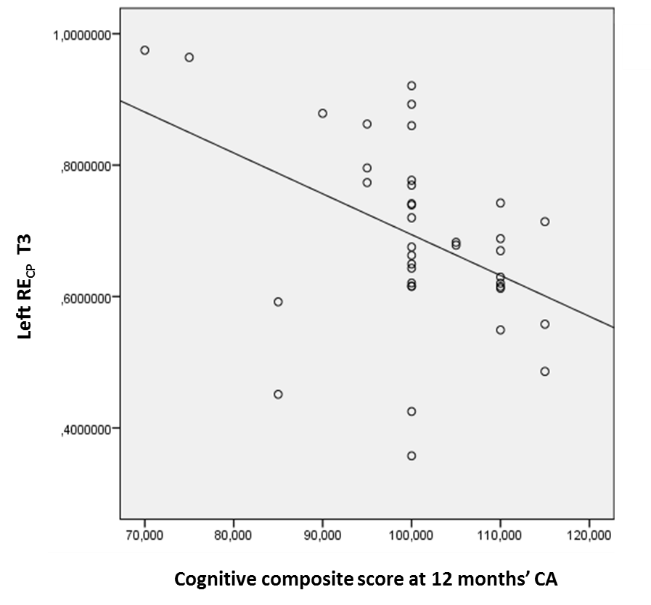

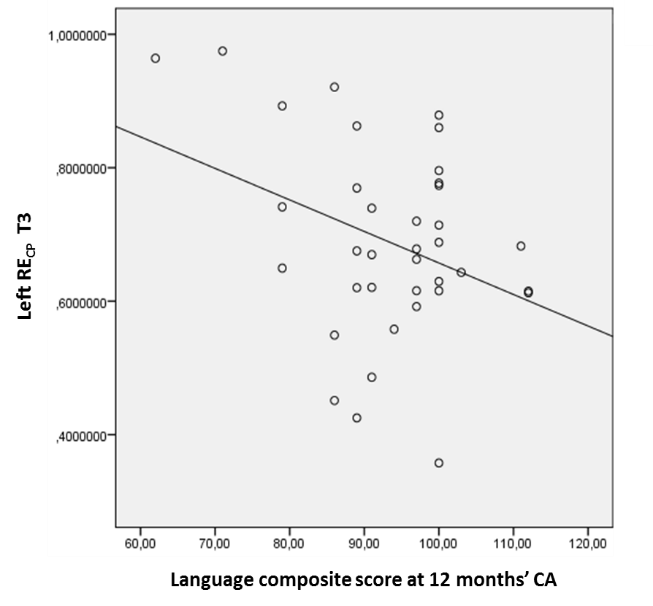


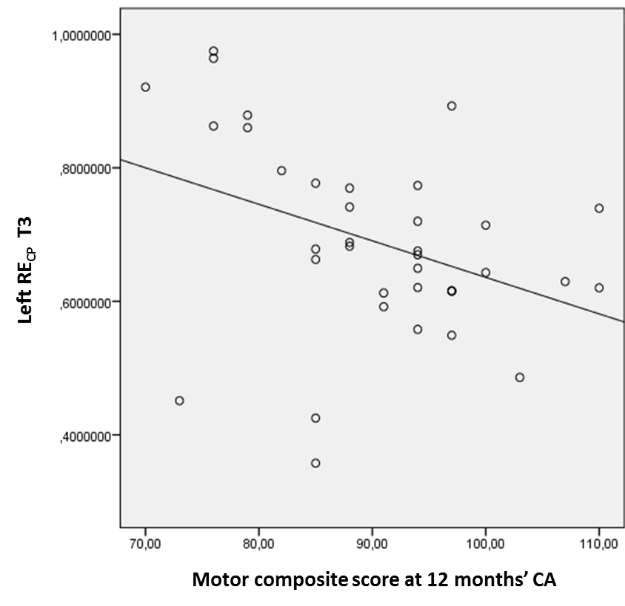


Figure S4. Significant correlations between RE_CP_ values from both right and left parasagittal scans at T_3_ and neurodevelopmental composite scores at 12 months’ CA. Cognitive composite score: Right RE_CP_ (*r*= -0.332, *p*=0.042) and Left RE_CP_ (*r*= -0.433, *p*=0.007). Language composite score: Right RE_CP_ (*r*= -0.521, *p*=0.001) and Left RE_CP_ (*r*= -0.344, *p*=0.037). Motor composite score: Left RE_CP_ (*r*= -0.375, *p*=0.022).
