# Supplementary material for: Quantitative ultrasonographic examination of cerebral white matter by pixel brightness intensity as marker of middle-term neurodevelopment: a prospective observational study
Source: Sci Rep. 2023 Oct 5;13:16816. doi: 10.1038/s41598-023-44083-w (PMC10556025; doi:10.1038/s41598-023-44083-w)
Supplement: Supplementary file 5 — Supplementary Table S1. [file 41598_2023_44083_MOESM5_ESM.docx]

Table s1. Percentage of patients with pathological right and left RE_CP_ values at T_0_, T_1_, T_2_, and T_3_ showing cognitive composite scores <85 or ≥85 at 12 months’ corrected age.

|  | | | Pathological cognitive composite score^b^ | Normal cognitive composite score^c^ | p-value |
| --- | --- | --- | --- | --- | --- |
| Pathological RE_CP_^a^ | T_0_ | Right | 1/3 (33.3%) | 10/40 (25.0%) | 0.598 |
|  |  | Left | 1/3 (33.3%) | 10/40 (25.0%) | 0.598 |
|  | T_1_ | Right | 2/3 (66.7%) | 8/38 (21.1%) | 0.142 |
|  |  | Left | 2/3 (66.7%) | 8/38 (21.1%) | 0.142 |
|  | T_2_ | Right | 2/3 (66.7%) | 6/28 (21.4%) | 0.156 |
|  |  | Left | 3/3 (100.0%) | 5/28 (17.9%) | 0.012* |
|  | T_3_ | Right | 2/2 (100.0%) | 8/36 (22.2%) | 0.064 |
|  |  | Left | 2/2 (100.0%) | 8/36 (22.2%) | 0.064 |

Legend: ^a^, RE_CP_ value ≥75^th^ percentile; ^b^, cognitive composite score <85 at 12 months’ corrected age; ^c^, cognitive composite score ≥85 at 12 months’ corrected age; *, statistically significant (p <0.05); T_0_, 0-7 days of life; T_1_, 14-35 days of life; T_2_, 37^0/7^-41^6/7^ weeks’ postmenstrual age; T_3_, 42^0/7^-52^0/7^ weeks’ postmenstrual age.
