# Supplementary material for: Quantitative ultrasonographic examination of cerebral white matter by pixel brightness intensity as marker of middle-term neurodevelopment: a prospective observational study
Source: Sci Rep. 2023 Oct 5;13:16816. doi: 10.1038/s41598-023-44083-w (PMC10556025; doi:10.1038/s41598-023-44083-w)
Supplement: Supplementary file 7 — Supplementary Table S3. [file 41598_2023_44083_MOESM7_ESM.docx]

Table s3. Percentage of patients with pathological right and left RE_CP_ values at T_0_, T_1_, T_2_, and T_3_ showing motor composite scores <85 or ≥85 at 12 months’ corrected age.

|  | | | Pathological motor composite score^b^ | Normal motor composite score^c^ | p-value |
| --- | --- | --- | --- | --- | --- |
| Pathological RE_CP_^a^ | T_0_ | Right | 4/9 (44.4%) | 7/34 (20.6%) | 0.201 |
|  |  | Left | 4/9 (44.4%) | 7/34 (20.6%) | 0.201 |
|  | T_1_ | Right | 6/9 (66.7%) | 4/31 (12.9%) | 0.003* |
|  |  | Left | 7/9 (77.8%) | 2/31 (6.5%) | 0.000* |
|  | T_2_ | Right | 3/7 (42.9%) | 5/23 (21.7%) | 0.345 |
|  |  | Left | 6/7 (85.7%) | 2/23 (8.7%) | 0.000* |
|  | T_3_ | Right | 6/8 (75.0%) | 4/29 (13.8%) | 0.002* |
|  |  | Left | 7/8 (87.5%) | 3/29 (10.3%) | 0.000* |

Legend: ^a^, RE_CP_ value ≥75^th^ percentile; ^b^, motor composite score <85 at 12 months’ corrected age; ^c^, motor composite score ≥85 at 12 months’ corrected age; *, statistically significant (p <0.05); T_0_, 0-7 days of life; T_1_, 14-35 days of life; T_2_, 37^0/7^-41^6/7^ weeks’ postmenstrual age; T_3_, 42^0/7^-52^0/7^ weeks’ postmenstrual age.
