# Supplementary material for: Quantitative ultrasonographic examination of cerebral white matter by pixel brightness intensity as marker of middle-term neurodevelopment: a prospective observational study
Source: Sci Rep. 2023 Oct 5;13:16816. doi: 10.1038/s41598-023-44083-w (PMC10556025; doi:10.1038/s41598-023-44083-w)
Supplement: Supplementary file 8 — Supplementary Table S4. [file 41598_2023_44083_MOESM8_ESM.docx]

Table s4. Correlations between RE_CP_ values from both right and left parasagittal scans and neurodevelopmental composite scores at 12 months’ CA.

|  | | Cognitive composite score at 12 months’ CA | Language composite score at 12 months’ CA | Motor composite score at 12 months’ CA |
| --- | --- | --- | --- | --- |
| T_0_ | Right RE_CP_ | r= 0.120 (p=0.445) | r= 0.120 (p=0.444) | r= -0.098 (p=0.532) |
|  | Left RE_CP_ | r= -0.112 (p=0.475) | r= 0.067 (p=0.669) | r= -0.219 (p=0.158) |
| T_1_ | Right RE_CP_ | **r= -0.380 (p=0.014)*** | **r= -0.345 (p=0.029)*** | **r= -0.382 (p=0.015)*** |
|  | Left RE_CP_ | **r= -0.471 (p=0.002)*** | r= -0.270 (p=0.092) | **r= -0.515 (p=0.001)*** |
| T_2_ | Right RE_CP_ | **r= -0.405 (p=0.024)*** | **r= -0.496 (p=0.005)*** | r= -0.287 (p=0.124) |
|  | Left RE_CP_ | **r= -0.460 (p=0.009)*** | r= -0.355 (p=0.054) | **r= -0.373 (p=0.042)*** |
| T_3_ | Right RE_CP_ | **r= -0.332 (p=0.042)*** | **r= -0.521 (p=0.001)*** | r= -0.312 (p=0.060) |
|  | Left RE_CP_ | **r= -0.433 (p=0.007)*** | **r= -0.344 (p=0.037)*** | **r= -0.375 (p=0.022)*** |

CA, corrected age; RE_CP_, echogenicity of parieto-occipital periventricular white matter relative to homolateral choroid plexus; T_0_, 0-7 days of life; T_1_, 14-35 days of life; T_2_, 37^0/7^-41^6/7^ weeks’ postmenstrual age; T_3_, 42^0/7^-52^0/7^ weeks’ postmenstrual age; *, statistically significant
